# Effect of Loading and Heating History on Deformation of LaCoO_3_

**DOI:** 10.3390/ma14133543

**Published:** 2021-06-25

**Authors:** Mykola Lugovy, Dmytro Verbylo, Nina Orlovskaya, Michael Reece, Jakob Kuebler, Thomas Graule, Gurdial Blugan

**Affiliations:** 1 Institute for Problems of Materials Science, 03142 Kyiv, Ukraine; ver@ipms.kiev.ua (M.L.); ver@materials.kiev.ua (D.V.); 2 Department of Mechanical and Aerospace Engineering, University of Central Florida, Orlando, FL 32816, USA; 3 School of Engineering and Materials Science, Queen Mary University of London, London E1 4NS, UK; m.j.reece@qmul.ac.uk; 4 Empa, Swiss Federal Laboratories for Materials Science and Technology, Laboratory for High Performance Ceramics, 8600 Duebendorf, Switzerland; jakob.kuebler@gmail.com (J.K.); Thomas.Graule@empa.ch (T.G.); Gurdial.Blugan@empa.ch (G.B.)

**Keywords:** lanthanum cobaltite, strength, high temperature, ferroelasticity, loading history

## Abstract

The aim of this work was to study cyclic stress–strain deformation behavior of LaCoO_3_ as a function of loading and heating history. The ferroelastic hysteretic deformation of LaCoO_3_ at different stresses and temperatures was characterized using effective Young’s modulus, hysteresis loop area and creep strain shift parameters. The deformation behavior of LaCoO_3_ was not significantly affected by the previous loading and heating history when tested at constant temperature. The high temperature strength and Young’s modulus of LaCoO_3_ were higher compared to at room temperature. A creep strain shift parameter was introduced to characterize creep strain in LaCoO_3_ for the first time.

## 1. Introduction

The deformation behavior of LaCoO_3_-based ceramics has been studied in great detail in the past [1,2,3,4,5,6,7,8]. The bulk modulus (~122 GPa) was extrapolated from high pressure changes in the lattice parameters of LaCoO_3_ [8]. While it was noticed that the reported bulk modulus of LaCoO_3_ has a lower value at RT as compared to PrCoO_3_ (~168 GPa) and NdCoO_3_ (~165 GPa) perovskites [8], the reported values are not consistent with Young’s modulus values of LaCoO_3_ reported in [9]. It is well established that unlike many other ceramics, such as B_4_C, Si_3_N_4_, SiC, or Al_2_O_3_, LaCoO_3_-based perovskites exhibit nonlinearity, ferroelasticity, and hysteresis upon mechanical loading [1,2]. Such non-linear hysteretic behavior is responsible for the appearance of permanent irreversible deformation and, thus, limits the use of LaCoO_3_ perovskites to applications where the applied stresses are minimal or non-existent. It was also established that pure LaCoO_3_ exhibits a significant stiffening during deformation at elevated temperature, such that its Young’s modulus increases from ~70 GPa upon loading at room temperature to ~120 GPa upon loading at 800 °C [7]. An attempt to explain the unusual high temperature stiffening by a possible high temperature phase transition of the LaCoO_3_ crystal lattice was not fruitful, as high-resolution neutron diffraction clearly showed that the crystal structure of LaCoO_3_ remains $R\bar{3}c$ rhombohedral and only a thermal expansion of the lattice was detected upon heating. It was not clear how an expanding lattice with associated increasing bond lengths upon heating could contribute to a significant increase of the Young’s modulus and stiffening of LaCoO_3_. However, when LaCoO_3_ is doped with 20 at % Ca on the La site of the perovskite lattice, the stiffening effect disappears, and La_0.8_Ca_0.2_CoO_3_ perovskite exhibits the expected high temperature softening behavior due to thermal expansion, and associated increased lattice parameters and unit cell volume [7,9].

The elevated temperature deformation behavior of LaCoO_3_ and La_0.8_Ca_0.2_CoO_3_ were studied on loading/unloading of the perovskite samples to different stresses in four-point bending [7,10]. It is well known that for ferroelastic ceramics, the loading history has a significant effect on their mechanical behavior [11]. Therefore, in some of the high temperature experiments [7], the LaCoO_3_ samples were loaded to a very small stress of 8 MPa in four-point bending in order to minimize the loading history effect on their stress–strain hysteresis loops. Even with loading at such a low stress level, the changes in loading/unloading hysteresis loops were significant during elevated temperature testing, and stiffening and decrease in hysteresis loop area were reported for pure LaCoO_3_ in the 700–900 °C temperature range [7]. Indeed, loading LaCoO_3_ to a higher stress level, such as 54 MPa [10], the deformation behavior detected upon loading to 8 MPa stress level was repeated. It was determined that loading to both 8 MPa and 54 MPa softens LaCoO_3_ in the 300–500 °C temperature range, where a slight decrease in Young’s modulus is observed, however, its stiffness increases significantly during cycling at 700–900 °C. The softening at 400 °C was accompanied by an increase in the hysteresis, but at 700–900 °C the hysteresis decreased significantly in comparison to the room temperature experiments. The stress–strain deformation became significantly more linear at 700 °C and especially at 800 °C, accompanied by an increase in the Young’s modulus of pure LaCoO_3_.

The high temperature first order phase transition in LaCoO_3_ was reported in [12]. However, in [13] it was reported that the first order phase transition at ~900 °C might occur because of the presence of Co_3_O_4_ secondary phase, which precipitates in Co rich regions of LaCoO_3_ upon heating. Co_3_O_4_ spinel has a higher Young’s modulus of 218 GPa [14] and if present might produce a detectable increase in Young’s modulus. However, to have such a significant increase as reported in [7,10], a significant amount of Co_3_O_4_ (up to 10 vol%) should precipitate upon heating and loading of LaCoO_3_. No evidence of such precipitation exists at the moment and in situ neutron diffraction has to be performed upon loading of LaCoO_3_ at 700–900 °C to verify the hypothesis.

It was determined that not only the loading history, but also the heating history, affects the deformation behavior of this perovskite. As such, experiments were performed that after loading at a high temperature, the sample was cooled down and tested again to the same stress level (54 MPa) at room temperature. As a result of these experiments, it was determined that the material that was exposed to the cyclic bending at 700 °C, 800 °C, and 900 °C becomes more and more hysteretic and compliant at room temperature as the temperature of the preceding high temperature experiments was increased. These high temperature and room temperature experiments indicate that not only the loading history, but also a previous high temperature exposure, plays an important role in the deformation behavior of LaCoO_3_. It was determined that after bending experiments performed at high temperature, the corresponding room temperature cyclic loading showed changes in mechanical behavior of LaCoO_3_ affecting its Young’s modulus, hysteresis loop area, and irreversible strain [10]. A comparison of LaCoO_3_ hysteretic behavior at the same temperature of mechanical testing after different heating history was not performed in the previous studies, and the objective of the current research is to fill this gap in our knowledge.

In the current research, a comparison of elevated and room temperature deformation of LaCoO_3_ is performed, and the stress–strain deformation curves of LaCoO_3_ all the way to failure at room temperature and 800 °C are presented.

## 2. Materials and Methods

**Figure 1:**
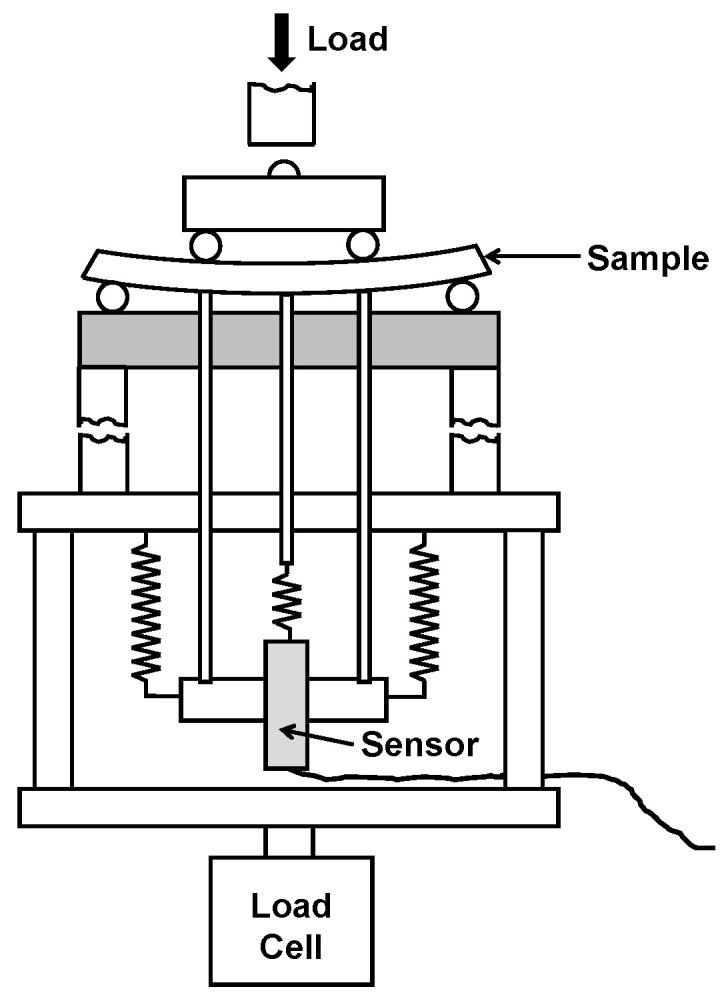
A schematic presentation of a 4-point bending set up. The load is applied by a 2 kN load cell and the deflection is measured by a 3-rod measurement system, where a sensor collects deflection data directly from the sample’s surface under tension.

The LaCoO_3_ perovskites were sintered by Praxair Surface Technologies, Specialty Ceramics, Manchester, CT, USA. Bars of 2.5 mm × 4 mm × 50 mm nominal dimensions were machined by PremaTech Ceramics, Worcester, MA, USA. Four-point bending experiments were performed on three LaCoO_3_ bending bars numbered as samples #1, #2, and #3 using a bending jig with 5 mm rollers, 40 mm supporting and 20 mm loading spans, and a 2 kN load cell on a universal testing machine (Zwick, Ulm, Germany) [15]. The tests were performed in load control mode with a loading/unloading rate of 1 N/s. The samples were preloaded to 10 N, which was set up as a zero point for further loading. From this zero-load set up point, the samples #1 and #2 were cyclically loaded to 10 N, 20 N, 30 N, and 40 N, equivalent to a maximum cyclic stress (σ_max_) of 13.5 MPa, 27 MPa, 40.5 MPa, and 54 MPa, respectively, thus four loading/unloading cycles were performed for each experiment at relevant temperatures. Sample #3 was cyclically loaded to 10 N, 20 N, 30 N, 40 N, 50 N, and 60 N load or σ_max_ equal to 13.5 MPa, 27 MPa, 40.5 MPa, 54 MPa, 67.5 MPa, and 81 MPa, respectively. Therefore, a total of six loading/unloading cycles were performed on this sample. The description of the heating history of all three cyclically-loaded LaCoO_3_ samples is presented in Table 1. While sample #1 of LaCoO_3_ was not broken at the end of the experiment, sample #2 of LaCoO_3_ failed during RT cycling and sample #3 of LaCoO_3_ failed during cyclic loading at 800 °C cycling. The high temperature cycling experiments were performed using a high temperature furnace (Maytec, Singen, Germany) with a heating rate of 15 °C/min and dwell time of 20 min before the cyclic experiments in order to equilibrate the samples. A 3-point deflection measurement system (Maytec, Singen, Germany) was used to measure the displacement of the samples both at room and high temperatures, where the deflection of the specimens was recorded by the central rod of a deflectometer positioned at the tensile surface of the samples and two control rods were positioned below the loading rollers at a distance of 10 mm from both sides of the central rod (Figure 1). The elastic beam equation was used calculate the stress on the tensile surface under bending of the LaCoO_3_ bars, which gives a slightly overestimated values of true stress due to the nonlinear stress–strain behavior of LaCoO_3_ [8,9], however, it was shown that such overestimation is not significant. The Young’s modulus obtained from the stress–strain deformation plots were calculated from the secant modulus of a straight line approximated at zero applied stress. It was reported [7] that such calculations correlate very well with the Young’s modulus values obtained by different techniques, such as impulse excitation, uniaxial compression, or resonant ultrasound spectroscopy.

## 3. Results and Discussion

### 3.1. Loading and Heating History of LaCoO_3_ Cycling

**Figure 2:**
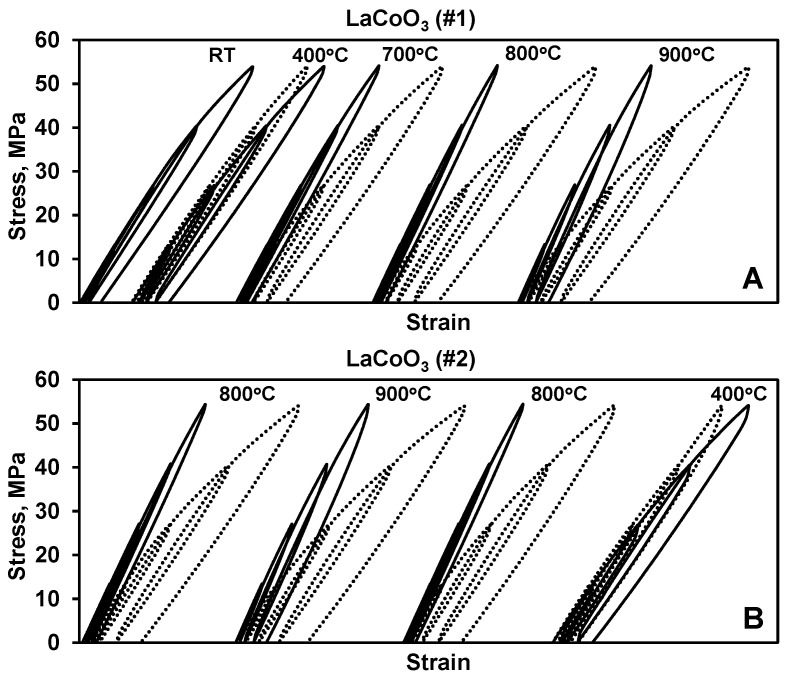
Stress–strain deformation plots of LaCoO_3_ sample #1 (**A**) and sample #2 (**B**) showing the corresponding heating history of the cyclic loading. The stress–strain plots performed at elevated temperatures are shown using a solid line, while room temperature stress–strain deformation plots collected after corresponding high temperature experiments are shown with a dotted line.

The stress–strain deformation behavior of two different LaCoO_3_ samples as a function of temperature and specific loading cycles is shown in Figure 2. LaCoO_3_ sample #1 was first cyclic loaded at RT. The Young’s moduli and hysteresis loop areas calculated using the obtained stress–strain hysteresis plots of this sample are shown in Table 2. It was determined that RT Young’s modulus and hysteresis loop area of the first cycle in this test were equal to 67 GPa and 0.26 kPa, respectively. The sample was then heated and the same four step cyclic loading was performed at 400 °C. It was determined that the LaCoO_3_ softened slightly and hysteresis loop areas became bigger for the test at 400 °C as compared to the original RT tests. The Young’s modulus of LaCoO_3_ decreased to 65 GPa as compared to 67 GPa at RT, but the hysteresis loop area almost doubled from 0.26 kPa at RT to 0.43 kPa at 400 °C in the first cycle. However, when comparing the fourth cycle, the Young’s moduli were calculated as 67 GPa at RT and 62 GPa at 400 °C, while the hysteresis loop areas remained almost the same and were equal to 6.3 kPa and 6.9 kPa for RT and 400 °C, respectively. After cycling at 400 °C, the sample was cooled down and cycled again at RT. The Young’s modulus became 66 GPa and the hysteresis loop areas decreased to 5.2 kPa as measured in the fourth cycle upon loading. As one can see from the above results, the heating and loading history did not affect the RT deformation behavior of LaCoO_3_ after testing at 400 °C. If LaCoO_3_ was a typical ceramic material, it would be expected that the softening would continue for further deformation at higher temperatures, however, since LaCoO_3_ is a unique perovskite, the stress–strain deformation behavior was distinctively different for this composition at 700 °C, 800 °C, and even 900 °C. In this temperature range, the hysteresis loop areas became much smaller, especially in the 800 °C test as compared to the 400 °C test. The Young’s modulus of LaCoO_3_ increased significantly and become equal to 73 GPa, 83 GPa, and 87 GPa at 700 °C, 800 °C, and 900 °C in the first cycle, respectively (Table 2). Despite being stiffer and less hysteretic upon deformation at 700–900 °C temperature range, after cooling down, the RT cyclic deformation cycling of LaCoO_3_ showed increased hysteresis loop areas and significant RT softening. For example, in the fourth cycle the hysteresis loop area increased from 3.3 kPa at 700 °C to 8.6 kPa at RT cycling, from 2.6 kPa at 800 °C to 10.2 kPa at RT, and from 5.1 kPa at 900 °C to 11.6 kPa at RT. At the same time, the Young’s modulus, calculated from the fourth stress–strain hysteresis loop, decreased from 75 GPa at 700 °C to 66 GPa at RT, from 83 GPa at 800 °C to 65 GPa at RT, and from 91 GPa at 900 °C to 64 GPa at RT (Table 2).

Unlike the first LaCoO_3_ sample, the second LaCoO_3_ sample was not tested at RT initially, but, instead, the stress–strain deformation plots were collected at 800 °C (Figure 2B). After cycling at 800 °C, the sample was cooled down and the same loading/unloading test was repeated again at RT. Similar to sample #1, LaCoO_3_ showed significant stiffening and decrease in hysteresis loop areas at 800 °C, such that its Young’s modulus was equal to 83 GPa at 800 °C, but softened significantly during the consequent RT test, where the Young’s modulus was measured to be equal to only 64 GPa. The hysteresis loop areas increased from 2.6 kPa at 800 °C to 10.5 kPa at RT. After cycling at 800 °C and consequent RT, the LaCoO_3_ sample #2 was heated again to 900 °C, cooled down and cycled again at RT. This heating and loading history was followed by heating to 800 °C one more time, where LaCoO_3_ exhibited the stiffening again, followed by the softening upon cooling and cycling at RT. The final elevated temperature cycling test of this LaCoO_3_ sample was performed at 400 °C, after which the sample was cooled down and tested at RT. The Young’s moduli and hysteresis loop areas of both LaCoO_3_ samples tested in these experiments are summarized in Table 2.

### 3.2. Stress–Strain Deformation Behavior of Different LaCoO_3_ Samples Tested at Identical Conditions

**Figure 3:**
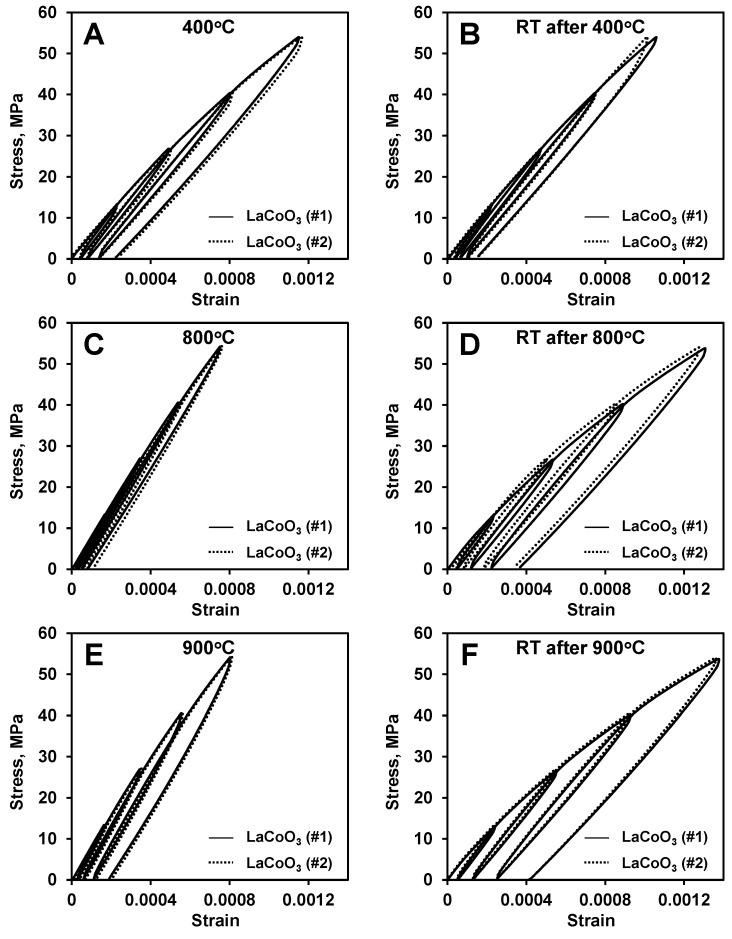
A comparison of different heating history stress–strain deformation plots of LaCoO_3_ sample #1 and sample #2 at: (**A**) 400 °C; (**B**) RT after 400 °C; (**C**) 800 °C; (**D**) RT after 800 °C; (**E**) 900 °C; (**F**) RT after 900 °C.

A comparison of the stress–strain diagram of two different LaCoO_3_ samples tested at different temperatures is shown in Figure 3. As one can see from Figure 3A, at 400 °C, the stress–strain diagrams collected from the two different samples coincide very well, despite totally different loading and heating histories preceding the cycling testing at 400 °C (Table 1). The Young’s moduli as well as hysteresis loop areas, measured from all four cycles during these tests are almost identical (Table 2). For example, the Young’s modulus measured using cycle 4 stress–strain data was equal to 62 GPa both for sample #1 and sample #2, despite the samples experiencing very different heating histories before these experiments. Upon cooling after cyclic loading at 400 °C, the stress–strain deformation behavior of these two LaCoO_3_ samples were also almost identical for cyclic tests performed at RT (Figure 3B), as the Young’s moduli were measured to be 66 GPa and 65 GPa and hysteresis loop areas was measured to be 5.2 kPa and 5 kPa in the 4th cycle for the first and second samples of LaCoO_3_, respectively.

Similar to the good reproducibility of the hysteretic behavior of LaCoO_3_ at 400 °C regardless of previous loading and heating histories, the very same phenomenon was observed for testing performed at 800 °C (Figure 3C) and following RT (Figure 3D) stress–strain deformations. As one can see from Figure 3C, while the hysteresis loop areas are much smaller for tests performed at 800 °C as compared to 400 °C (Figure 3A), despite the very different loading and heating history of these samples preceding the cyclic testing at 800 °C (Table 1), the Young’s moduli as well as hysteresis loop areas measured for all four cycles during these tests are almost identical (Table 2). For example, the Young’s modulus measured at 800 °C using the fourth cycle stress–strain data was equal to 83 GPa for the first sample and 83 GPa and 87 GPa for the second sample, which was cycled twice at 800 °C (Table 1). The difference in 4 GPa between first and second cycling at 800 °C for the LaCoO_3_ sample #2 is very small and could potentially be neglected in this study. The hysteresis loop areas measured from the fourth cycle at 800 °C were 2.6 kPa for the first LaCoO_3_ sample and 2.4 kPa for the second LaCoO_3_ sample for first cycling, which are all very similar to each other (Table 2). While, three cycling tests were performed at 800 °C on two LaCoO_3_ samples (Table 1 and Figure 2), for clarity only two of these stress–strain deformation plots are shown in Figure 3C. One of them was taken from testing of sample #1 and the other was taken from first cycling at 800 °C of sample #2. Upon cooling after 800 °C, the stress–strain deformation behavior of these LaCoO_3_ samples were also almost identical for cyclic tests performed at RT (Figure 3D), with Young’s modulus measured to be equal to 65 GPa for the first sample and 65 GPa and 64 GPa for two tests performed on the second LaCoO_3_ sample. Only two stress–strain deformation diagrams are also shown in Figure 3D—one is at RT after cycling at 800 °C of the first LaCoO_3_ sample and a second one is at RT after the first 800 °C cycling of the second LaCoO_3_ sample. The hysteresis loop areas measured from the fourth cycle at RT after 800 °C testing were equal to 10.2 kPa for the first LaCoO_3_ sample and 10.5 kPa and 10 kPa for the second LaCoO_3_ sample, which are almost four times larger as compared to the loop areas measured at 800 °C.

The same similarities were observed when the comparison of the hysteretic behavior of LaCoO_3_ perovskite was made for results performed at 900 °C (Figure 3E) and subsequent RT (Figure 3F) stress–strain deformation behavior. As one can see from Figure 3E, the hysteresis loop areas measured at 900 °C increased in comparison with the tests performed at 800 °C. However, both LaCoO_3_ samples exhibited very similar deformation behaviors, despite different loading and heating histories, where the measured Young’s moduli and hysteresis loop areas were almost identical for the two samples cycled at 900 °C and then RT (Table 2). Thus, the Young’s modulus measured at 900 °C for the 4th cycle was equal to 91 GPa and 92 GPa and the hysteresis loop areas were equal to 5.1 kPa and 5.2 kPa for the first and second samples, respectively. Upon cooling after 900 °C, the stress–strain deformation behavior of these LaCoO_3_ samples were again almost identical between each other for the cyclic tests performed at RT (Figure 3F). For these RT cycling, the Young’s moduli were equal to 64 GPa and 65 GPa for the first and second samples respectively, and the hysteresis loop areas were measured to be equal to 11.6 kPa for both samples as measured from the fourth cycle at RT after 900 °C testing.

### 3.3. Stress–Strain Deformation Behavior of LaCoO_3_ Tested at Different Conditions

**Figure 4:**
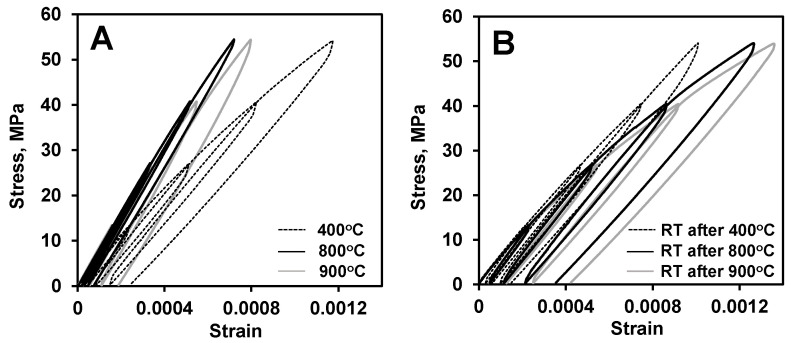
A comparison of stress–strain plots of LaCoO_3_ sample #2 tested (**A**) at elevated and (**B**) at room temperature. LaCoO_3_ sample tested at 900 °C (**A**) experienced the previous loading at 800 °C and RT, at 800 °C (**A**) experienced the previous loading at 800 °C, RT, 900 °C, and RT, and at 400 °C (**A**) experienced the previous loading at 800 °C, RT, 900 °C, RT, 800 °C, and RT. The same LaCoO_3_ sample tested at RT (**B**) experienced the 800 °C and RT previous loading when tested at RT after 900 °C; 800 °C, RT, 900 °C, and RT previous loading when tested at RT after 800 °C; and 800 °C, RT, 900 °C, RT, 800 °C and RT previous loading when tested at RT after 400 °C.

**Figure 5:**
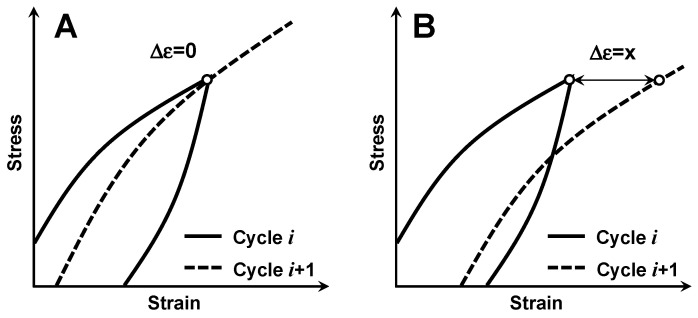
A schematic presentation of cyclic stress–strain deformation behavior indicating absence of creep strain shift Δε = 0 (**A**) and presence of creep strain shift Δε = x (**B**).

**Figure 6:**
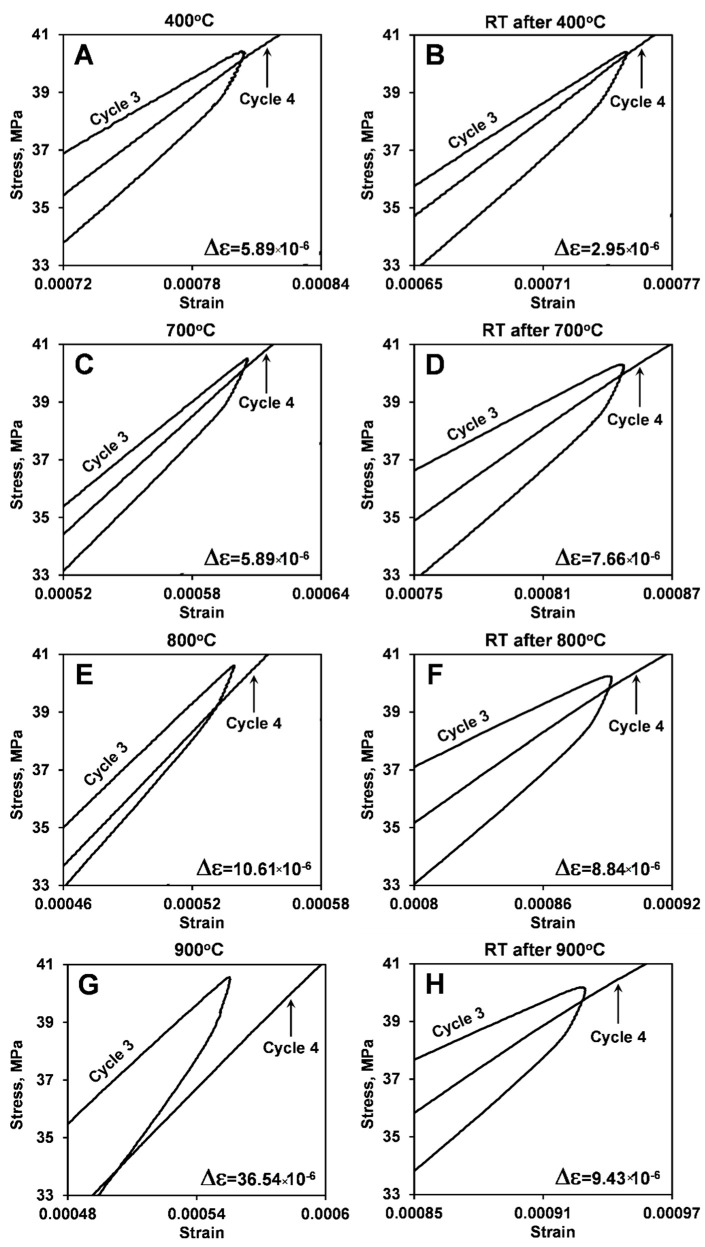
Portions of cycle 3 loading/unloading and cycle 4 loading stress-strain deformation plots of LaCoO_3_ sample #1 showing the presence of creep strain shift Δε at testing performed at different temperatures: (**A**) 400 °C; (**B**) RT after 400 °C; (**C**) 700 °C; (**D**) RT after 700 °C; (**E**) 800 °C; (**F**) RT after 800 °C; (**G**) 900 °C; (**H**) RT after 900 °C. The corresponding values of Δε are shown in Table 3.

A comparison of selected stress–strain deformation curves obtained at different temperatures and heating histories of LaCoO_3_ sample #2 is show in Figure 4. The three stress–strain deformation plots collected at 400 °C, 800 °C, and 900 °C are shown in Figure 4A. The three corresponding stress–strain deformation plots collected at RT after cooling the sample after testing at 400 °C, 800 °C, and 900 °C are shown in Figure 4B. As one can see from Figure 4A, the most non-linear deformation upon loading of LaCoO_3_ occurred at 400 °C, where the Young’s modulus and hysteresis loop area were calculated from the fourth cycle to be equal to 62 GPa and 7.9 kPa, respectively. When the temperature of the test increased to 800 °C, LaCoO_3_ exhibited much more elastic like behavior, as compared to the stress–strain deformation exhibited at 400 °C. The material stiffened significantly, with the Young’s modulus measured from the fourth cycle to be equal to 87 GPa and corresponding hysteresis loop area decreased from 7.2 kPa at 400 °C to 2.4 kPa at 800 °C. The increase of the cycling temperature to 900 °C brings an increase in hysteresis loop areas and deviation from linearity, as compared to 800 °C, however, such an increase and more non-linear stress–strain deformation behavior occurs because of the presence of the high temperature creep (Figure 5 and Figure 6), while the Young’s modulus of LaCoO_3_ increased even further, as compared to 400 °C and 800 °C. The Young’s modulus of LaCoO_3_ at 900 °C measured from fourth cycle of stress–strain deformation curve was equal to 92 GPa and the corresponding hysteresis loop area increased from 2.4 kPa at 800 °C to 5.2 kPa at 900 °C. Thus, as one can see from Figure 4A, LaCoO_3_ behaves very different at 400 °C, 800 °C, and 900 °C and it exhibits a very unusual deformation behavior by showing less hysteretic and stiffer behavior at high temperature as compared to 400 °C test.

LaCoO_3_ sample #2 was further cycled under the same loading conditions upon cooling from each elevated temperature test, and the results of these RT cycling tests are shown in Figure 4B. As can see from Figure 4B, the RT deformation behavior of LaCoO_3_ is different and strongly depends on the proceeding high temperature cyclic test. Among these three selected high temperatures, the RT hysteresis loops had the smallest areas after testing at 400 °C, with a progressive increase in the hysteresis loop areas after testing at 800 °C and especially at 900 °C. For example, the RT hysteresis loop areas in the fourth cycles increased from 5 kPa measured after testing at 400 °C to 10 kPa measured after testing at 800 °C to 11.6 kPa after testing at 900 °C. However, despite such pronounced differences in hysteresis loop areas, the Young’s moduli of LaCoO_3_ measured for all three RT stress–strain deformation plots had very similar values and were equal to 65 GPa, 64 GPa, and 65 GPa at RT for tests performed after heating at 400 °C, 800 °C, and 900 °C, respectively.

### 3.4. Creep Strain Shift in LaCoO_3_ at Different Temperatures

One interesting feature of the deformation of LaCoO_3_, detected both at RT and elevated temperatures, was a small amount of creep present upon cyclic testing at each temperature. The presence of this creep was pronounced in the appearance of the creep strain shift Δε between the *i* and *i* + 1 loading/unloading cycles of the ceramics, as shown in the schematics of Figure 5. For the material, where creep is absent Δε = 0 (Figure 5A), and for the material, where creep is present Δε = x (Figure 5B). While creep, as a continuous deformation of a material with time, is traditionally reported to occur at temperatures higher than 0.5 *T_m_*, where *T_m_* is the melting temperature of the material, it was reported that LaCoO_3_ experiences RT creep [16]. Such RT creep is caused by the presence of highly mobile domain walls, stacking faults, oxygen point defects and complexes, as well as other defects in LaCoO_3_ grains [2,17]. These defects are able to move leading to the low stiffness of the cobaltite, causing a significant measurable creep deformation upon applied stress at RT. Creep is a very complex phenomenon, and creep strain is a function of stress, loading rate, time, temperature, grain size and morphology, presence of defects and their mobility, and other material parameters [18]. It was found that RT creep in LaCoO_3_ and high temperature creep in metals need to be described by a different set of equations. Ferroelastic creep in LaCoO_3_ results in an equilibrium saturation strain and zero strain rate at a given stress, which is attributed to ferroelastic domain switching [16]. However, high temperature primary creep in metals is followed by a constant nonzero strain rate—secondary creep, and further by tertiary creep with failure at the end. Because of such behavior, a different phenomenological approach has to be used to describe RT ferroelastic creep in LaCoO_3_ as compared to high temperature creep in metals [16].

While high temperature creep of La_0.5_Sr_0.5_Fe_1-x_Co_x_O_3-δ_ ceramics was studied in the 900–1050 °C temperature range in compression [17], no results for high temperature behavior of LaCoO_3_ have been reported. In this work, it was not originally intended to study high temperature creep deformation behavior, however, it was very easy to measure creep strain shift Δε during loading/unloading cycling of LaCoO_3_ at different temperatures, and therefore we would like to report our findings on Δε of LaCoO_3_.

**Figure 7:**
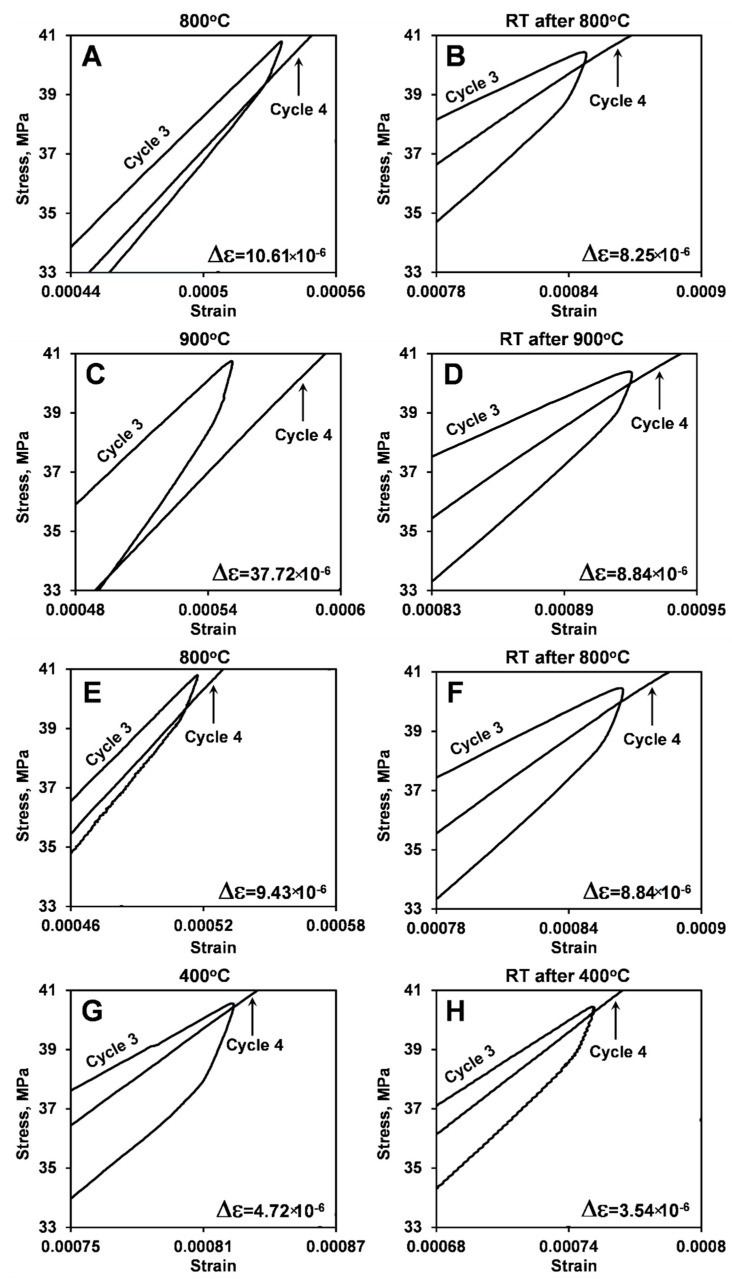
Portions of cycle 3 loading/unloading and cycle 4 loading stress-strain deformation plots of LaCoO_3_ sample #2 showing the presence of creep strain shift Δε at testing performed at different temperatures: (**A**) 800 °C; (**B**) RT after 800 °C; (**C**) 900 °C; (**D**) RT after 900 °C; (**E**) 800 °C; (**F**) RT after 800 °C; (**G**) 400 °C; (**H**) RT after 400 °C. The corresponding values of Δε are shown in Table 3.

The highlights of the stress–strain deformation plots between third loading/unloading and fourth loading cycles of the two different LaCoO_3_ samples at different temperatures are shown in Figure 6 and Figure 7. The corresponding Δε creep strain shifts are also presented in Figure 6 and Figure 7, and are summarized in Table 3. As one can see from Figure 6 and Figure 7, the creep strain shift in LaCoO_3_ increased from 2.95 × 10^−6^ at RT to 5.89 ×10^−6^ at 400 °C to 10.61 ×10^−6^ at 800 °C to 36.54 ×10^−6^ at 900 °C, which is a very significant and almost exponential increase with temperature. It is important to mention that Δε showed similar values for cycling both at 400 °C and 700 °C.

By comparing Δε measured from LaCoO_3_ samples #1 and #2, one can see that heating history does not significantly affect the creep strain shift at high temperatures, as the values of Δε are very similar for all three tests performed at 800 °C or two tests performed at 900 °C on two different samples. A very similar Δε = 10.73 ×10^−6^ was also found to occur between third and fourth cycles during testing of LaCoO_3_ samples #3.

The preceding high temperature cycling does have a significant effect on the room temperature deformation of LaCoO_3_. As one can see from Figure 6 and Figure 7, it is not only that the hysteresis loop areas increase but also the RT creep strain shift Δε also increased after high temperature cycling. Comparable Δε values were found for RT cycling of LaCoO_3_ after tests at 400 °C, 800 °C, and 900 °C (Table 3). Therefore, the measurements of creep strain shift Δε show that LaCoO_3_ experiences both RT and high temperature creep deformation, and it would be of interest to study temperature effect on creep of LaCoO_3_ in the future.

### 3.5. Four-Point Bending Strength of LaCoO_3_

**Figure 8:**
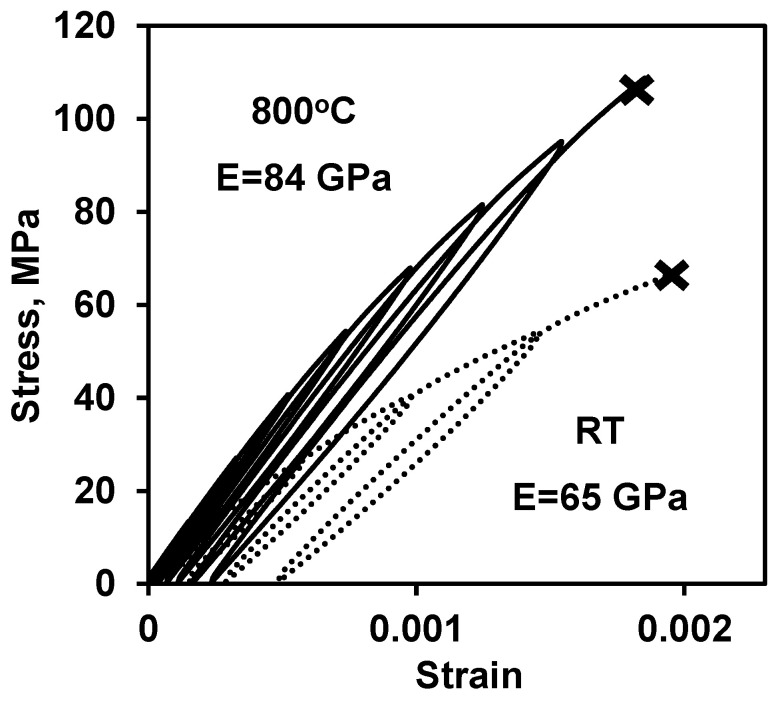
Four-point bending stress–strain deformation plots of LaCoO_3_ sample #2 at RT and sample #3 at 800 °C. The cross sign (**×**) indicates a moment of fracture of the samples.

**Figure 9:**
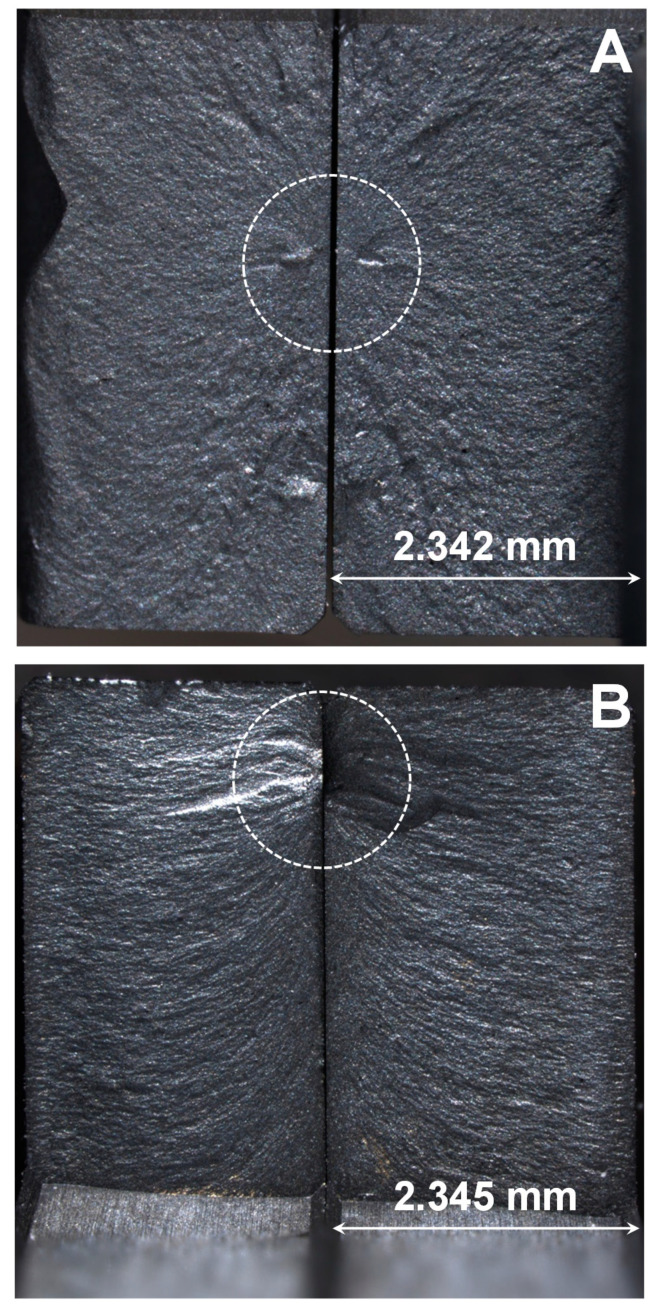
Optical micrographs of fracture surfaces of sample #2 failed at RT (**A**) and sample #3 failed at 800 °C (**B**).

Two LaCoO_3_ samples (#2 and 3) were loaded at RT and 800 °C all the way to failure in order to measure their strength (Figure 8). The strength of the LaCoO_3_ sample tested at RT was measured to be lower, as compared to the strength of the LaCoO_3_ sample tested at 800 °C. One of the explanations for this difference might be that the sample tested at RT had experienced a significant loading and heating history (Figure 2B) before this final test was performed. However, it was reported in the previous work [9,11,19] that the high temperature strength of LaCoO_3_ was indeed either on par or even higher as compared to strength measured at RT. For example, the RT strength of LaCoO_3_ was reported to be equal to 53 ± 2 MPa but at 850 °C it was measured to be 50 ± 5 MPa in [15]. In [8,10] the RT strength of LaCoO_3_ was reported to be 86 MPa and 72 ± 10 MPa, respectively, but at 800 °C, the strength of LaCoO_3_ was reported to be 109 ± 19 MPa in both papers. The LaCoO_3_ sample #2 failed after four RT loading/unloading cycles, the LaCoO_3_ sample #3 tested at 800 °C was able to withstand six loading/unloading cycles. Remarkably, all of the hysteresis parameters of the stress–strain deformation curves of the LaCoO_3_ sample #3 tested at 800 °C (Table 4) are in complete correspondence with results obtained after testing LaCoO_3_ samples #1 and #2 (Table 2). The optical micrographs of the fracture surfaces of the tested LaCoO_3_ sample #2 at RT and LaCoO_3_ sample #3 tested at 800 °C are shown in Figure 9A,B, respectively. The fracture origins, which are defects responsible for the initiation of failure, are highlighted with dashed circles in Figure 9. As one can see from Figure 9, both of the samples failed in a brittle manner, characteristic of the failure of most ceramic materials.

## 4. Conclusions

An attempt was made to understand the effect of loading and heating history on the ferroelastic hysteretic behavior of LaCoO_3_ perovskite. It was established that elevated temperatures have a significant effect on stress–strain deformation, hysteresis, and creep of LaCoO_3_ upon cycling, which is consistent with previously reported results. However, it was determined that the stress–strain deformation plots obtained at the same elevated temperature on different samples or with different cycling histories are almost identical, regardless of previous loading and heating histories. The parameters that describe the deformation behavior of LaCoO_3_, such as Young’s modulus, hysteresis loop area, and creep strain shift, were all confirmed to be very similar for the samples with different loading and heating histories tested at the same elevated temperature. Therefore, it was established that the previous history of loading and heating has no or very little effect on deformation behavior of LaCoO_3_ tested at the same elevated temperature.

The same congruence of the results has been determined to occur for the tests performed at RT after cycling at predetermined elevated temperature. It was found that the previous history of the LaCoO_3_ deformation did not affect the RT stress–strain behavior, however, the elevated temperature, which directly preceded the RT test, plays a tremendous role. The increase in the temperature of the mechanical test from 400 to 900 °C resulted in a significant increase in the hysteresis loop areas and creep strain shift of the stress–strain deformation curves. However, the RT Young’s modulus of LaCoO_3_ remains the same, regardless of the history of the previous elevated temperature tests.

According to the results obtained in this work, it is clear that at elevated temperatures the Young’s modulus increases and LaCoO_3_ became more linear-elastic as compared to its RT deformation behavior. However, as shown in the current work, LaCoO_3_ can withstand a higher number of loading/unloading cycles before complete failure at high temperature as compared to the RT cycling. The 4-point bending strength of LaCoO_3_ was determined to be higher in comparison with RT strength, similar to the increase in Young’s modulus.

## Figures and Tables

**Table 1:** Heating histories of three LaCoO_3_ samples.

Sample #	Temperatures of Cycling Loading
1	RT–400 °C–RT–700 °C–RT–800 °C–RT–900 °C–RT
2	800 °C–RT–900 °C–RT–800 °C–RT–400 °C–RT
3	800 °C

**Table 2:** Young’s moduli and hysteresis loop areas of two LaCoO_3_ samples calculated at four different loading/unloading cycles at different temperatures.

Sample #	T, °C	E, GPa				Hysteresis Loop Area, kPa			
		Cycle				Cycle			
		1	2	3	4	1	2	3	4
1	RT	67	69	68	67	0.26	0.71	2.15	6.30
	400	65	67	64	62	0.43	1.43	3.45	6.90
	RT	65	69	67	66	0.34	1.13	2.54	5.20
	700	73	75	76	75	0.22	0.70	1.61	3.30
	RT	66	69	67	66	0.46	1.82	4.32	8.60
	800	83	84	84	83	0.18	0.54	1.27	2.60
	RT	65	68	67	65	0.49	2.16	5.23	10.20
	900	87	90	90	91	0.24	0.94	2.45	5.10
	RT	64	67	67	64	0.52	2.30	5.79	11.60
2	800	83	84	84	83	0.18	0.54	1.27	2.60
	RT	64	68	67	65	0.32	1.53	5.24	10.50
	900	88	91	92	92	0.24	0.94	2.52	5.20
	RT	65	68	67	65	0.51	2.31	5.85	11.60
	800	85	87	87	87	0.18	0.52	1.17	2.40
	RT	66	71	68	64	0.50	2.08	5.12	10.00
	400	60	67	63	62	0.51	1.72	3.96	7.90
	RT	65	67	67	65	0.37	1.27	2.85	5.00

**Table 3:** The creep strain shift Δε of two LaCoO_3_ samples calculated at loading/unloading cycle 3 and loading cycle 4 at different temperatures.

LaCoO_3_ Sample #1			LaCoO_3_ Sample #2		
T, °C	Δε at T	Δε at RT after T	T, °C	Δε at T	Δε at RT after T
RT	2.95 ×10^−6^	-	-	-	-
400	5.89 ×10^−6^	2.95 ×10^−6^	800	10.61 ×10^−6^	8.25 ×10^−6^
700	5.89 ×10^−6^	7.66 ×10^−6^	900	37.72 ×10^−6^	8.84 ×10^−6^
800	10.61 ×10^−6^	8.84 ×10^−6^	800	9.43 ×10^−6^	8.84 ×10^−6^
900	36.54 ×10^−6^	9.43 ×10^−6^	400	4.72 ×10^−6^	3.54 ×10^−6^

**Table 4:** Young’s moduli, hysteresis loop areas and creep strain shift parameters characterizing stress–strain deformation behavior of LaCoO_3_ sample #3.

LaCoO_3_ Sample #3, 800 °C				
# Cycle	σ_max_, MPa	E, GPa	Hysteresis Loop Area, kPa	Creep Strain Shift Δε
1	13.50	84	0.18	3.54 ×10^−6^
2	27.00	85	0.56	5.89 ×10^−6^
3	40.50	84	1.33	10.73 ×10^−6^
4	54.00	83	2.74	15.32 ×10^−6^
5	67.50	82	5.01	22.40 ×10^−6^
6	81.00	81	8.26	31.83 ×10^−6^

## Data Availability

The data presented in this study are available on request from the corresponding authors.
